# Cisplatin Resistant Spheroids Model Clinically Relevant Survival Mechanisms in Ovarian Tumors

**DOI:** 10.1371/journal.pone.0151089

**Published:** 2016-03-17

**Authors:** Winyoo Chowanadisai, Shanta M. Messerli, Daniel H. Miller, Jamie E. Medina, Joshua W. Hamilton, Mark A. Messerli, Alexander S. Brodsky

**Affiliations:** 1 Department of Nutritional Sciences, Oklahoma State University, Stillwater, Oklahoma, United States of America, 74078; 2 Marine Biological Laboratory, Woods Hole, Massachusetts, United States of America, 02543; 3 Department of Biology, Massachusetts Institute of Technology, Cambridge, Massachusetts, United States of America, 02139; 4 Whitehead Institute for Biomedical Research, Cambridge, Massachusetts, United States of America, 02142; 5 Department of Biological Sciences, Bridgewater State University, Bridgewater, Massachusetts, United States of America, 02325; 6 Swenson College of Science and Engineering, University of Minnesota, Duluth, Minnesota, United States of America, 55804; 7 Department of Biology and Microbiology, South Dakota State University, Brookings, South Dakota, United States of America, 57007; 8 Department of Pathology and Laboratory Medicine, Rhode Island Hospital and Alpert Medical School of Brown University, Providence, Rhode Island, United States of America, 02903; 9 Center for Computational Molecular Biology, Brown University, Providence, Rhode Island, United States of America, 02912; Cedars-Sinai Medical Center, UNITED STATES

## Abstract

The majority of ovarian tumors eventually recur in a drug resistant form. Using cisplatin sensitive and resistant cell lines assembled into 3D spheroids we profiled gene expression and identified candidate mechanisms and biological pathways associated with cisplatin resistance. OVCAR-8 human ovarian carcinoma cells were exposed to sub-lethal concentrations of cisplatin to create a matched cisplatin-resistant cell line, OVCAR-8R. Genome-wide gene expression profiling of sensitive and resistant ovarian cancer spheroids identified 3,331 significantly differentially expressed probesets coding for 3,139 distinct protein-coding genes (Fc >2, FDR < 0.05) (S2 Table). Despite significant expression changes in some transporters including MDR1, cisplatin resistance was not associated with differences in intracellular cisplatin concentration. Cisplatin resistant cells were significantly enriched for a mesenchymal gene expression signature. OVCAR-8R resistance derived gene sets were significantly more biased to patients with shorter survival. From the most differentially expressed genes, we derived a 17-gene expression signature that identifies ovarian cancer patients with shorter overall survival in three independent datasets. We propose that the use of cisplatin resistant cell lines in 3D spheroid models is a viable approach to gain insight into resistance mechanisms relevant to ovarian tumors in patients. Our data support the emerging concept that ovarian cancers can acquire drug resistance through an epithelial-to-mesenchymal transition.

## Introduction

High Grade Serous Ovarian Cancer (HGSOC) is the most lethal form of ovarian cancer with approximately 16,000 new cases in the United States each year with 5 year survival rates <30% [1]. Platinum and taxane-based chemotherapy are the most common first-line agents, however, eventual resistance to cisplatin and recurrence of ovarian cancer following initial therapy is a major limitation [2], and is associated with poor prognosis following recurrence [3]. Thus, there is a pressing medical need to identify predictive markers in order to identify patients who will benefit from chemotherapy, and to develop new treatment options for this lethal disease.

Myriad mechanisms of platinum therapy resistance have been identified including changes in cisplatin transport and trafficking, disruption of apoptosis, increased tolerance of cisplatin-DNA adducts, and increased DNA repair in response to cisplatin-DNA interactions [4, 5]. Many tumors exhibit multiple resistance pathways simultaneously [6].

Experimental models have not recapitulated the many features exhibited in tumors including intercellular communication and the influence of the microenvironment [7]. There has been increasing interest in 3D culture models amenable for high-throughput screening [7, 8]. We aimed to characterize a spheroid cisplatin resistance model and determine how well this model recapitulates resistance mechanisms observed in patients. Moreover, more in vitro models are needed to model the extensive heterogeneity of HGSOC [7]. Other recently derived resistant models such as SKOV3 [9] may not be good models of HGSOC, [10] leaving just OVCAR3 as a potential model[11].

Towards these goals, we derived a new OVCAR-8 cisplatin resistant cell line (OVCAR-8R) and used genome wide expression analysis to discover genes differentially expressed in the sensitive and resistant cells as spheroids. Genes differentially expressed between the parental and resistant OVCAR-8 cells are enriched for markers of the mesenchymal state and are associated with survival. Despite significant expression changes of cisplatin transporters, OVCAR-8R spheroids did not show significantly different intracellular platinum concentration or transport properties compared to the parental OVCAR-8 spheroids. We applied multiple methods to evaluate how similar the expression changes adapted by OVCAR-8R may be reflected in HGSOC tumors. A pathway and a direct evaluation of a set of genes both indicated that many features of OVCAR-8R spheroids model HGSOC tumors. These data indicate that the OVCAR-8R spheroid model captured critical aspects of cisplatin resistance relevant to ovarian cancer patients.

## Materials and Methods

### Reagents

Cisplatin (cis-diamminedichloroplatinum(II)) was purchased from Sigma-Aldrich.

The human ovarian adenocarcinoma cancer cell line OVCAR-8 cell line was purchased through the National Cancer Institute Developmental Therapeutics Program’s tumor repository program. OVCAR-8 was made resistant *in vitro* by continuous stepwise exposure to cisplatin up to 5 μM to produce the corresponding cisplatin-resistant cell line OVCAR-8R. The cells were stably resistant after 6 weeks of growth in the absence of cisplatin. All cell lines were maintained in Dulbecco’s modified Eagle’s medium (DMEM) (Gibco, Grand Island, NY) containing 10% fetal bovine serum (FBS) with antibiotics (50 units penicillin/mL DMEM, 50 μg streptomycin/mL). Cells were grown as attached monolayers and incubated in a humidified atmosphere with 5% CO_2_ at 37°C. OVCAR-8 cell lines were authenticated by the ATCC Cell Line Authentication Service.

### Cell viability assay

Cells were plated in 96 well plates and treated 24 h later with the indicated concentrations of cisplatin. Viability was measured after 96 h of treatment using the WST-1 reagent (Roche), according to the manufacturer’s instructions.

### Spheroid generation

Spheroids were generated by seeding OVCAR-8 and OVCAR-8R cells in low attachment agarose gel molds with hemispherical recesses. Gel casts were created by pouring 2% agarose into 3D Petri Dish casting molds (Microtissues, Providence, RI) [12, 13]. To generate spheroids with a diameter of about 300 μm, approximately 10,000–25,000 cells in 190 μL DMEM were placed in each mold and allowed to grow for 2–3 days before collection.

### Cisplatin uptake assay

Net cisplatin uptake was determined by measuring platinum content of ovarian cancer cells before and after incubation with cisplatin. Equal numbers of OVCAR-8 and OVCAR-8R cells were plated in T75 flasks. Cells were treated with 5 μM cisplatin for 3 hrs. After cisplatin treatment, cells were washed once with HBSS, and then with PBS lacking calcium and magnesium. Cells were then dissociated in PBS containing no calcium or magnesium and containing 5 mM EDTA, and centrifuged at 229 x g. The pellet was dissolved in 20 mM Tris, pH 7.6 and the cells lysed by multiple freeze-thaw cycles at -80°C.

Platinum was measured by using an Agilent 7500CE ICP-MS (Agilent Technologies, Palo Alto, CA) at the Interdisciplinary Center for Plasma Mass Spectrometry at the University of California at Davis. Cell lysates and platinum standards were introduced using a MicroMist Nebulizer (Glass Expansion, Pocasset, MA) into a temperature controlled spray chamber. Platinum standard solutions, were diluted from standardized platinum stock solutions (SPEX CertiPrep, Metuchen, NJ) to concentrations from 0.01 to 300 ppb in 3% nitric acid (Fisher Scientific, trace metal grade) in deionized water (Millipore). Cisplatin content, calculated from platinum concentrations, was normalized to protein concentration (Bio-rad assay, Bio-rad. Hercules, CA), and background readings, derived as platinum content of cells prior to cisplatin incubation, were subtracted. Net cisplatin uptake (expressed as μg cisplatin/g protein) was first determined as mol platinum per g protein and converted to weight of cisplatin by multiplying by the ratio of molecular weights of cisplatin (300.1 g/mol) to platinum (195.1 g/mol).

### Gene expression profiling by microarray analysis

Total RNA was isolated from spheroids using the Trizol reagent (Invitrogen, Carlsbad, CA). Microarrays were processed at the Yale Center for Genome Analysis facility. RNA was fragmented and labeled with the Affymetrix GeneChip Whole Transcript Target Labeling Assay and hybridized to the Affymetrix Human Gene 1.0 ST Arrays according to recommended Affymetrix protocols (Affymetrix, Santa Clara, CA). Signals were calculated by Robust Multichip Analysis (RMA) using Expression Console software (Affymetrix, version 1.1). Genes with low signals, defined as the lowest quartile in both cisplatin sensitive and resistant cells, were excluded from further analysis. Raw and processed microarray data were deposited into the NCBI Gene Expression Omnibus database (GSE45553).

### Quantitative RT-PCR

Total RNA was isolated as described above and reverse-transcribed to cDNA as described previously [14]. Quantitative real-time PCR was performed using a Biorad iCycler (Hercules, CA) and PCR products were detected by EvaGreen-DNA binding (SsoFast EvaGreen Supermix, Biorad). Gene expression relative to glyceraldehyde 3-phosphate dehydrogenase (GAPDH) was determined by the ΔΔC_t_ method. Primer sequences are listed in S1 Table.

### Bioinformatics and survival analysis

The Significance Analysis of Prognostic Signatures (SAPs) code and ovarian tumor datasets were downloaded from dryad [15]. The OVCAR-8R derived datasets were added to the MSigDB gene set list for SAPs analysis.

Hierarchical clustering was performed in Gene-E using Pearson correlation to calculate distances [16]. Survival analysis including the Cox Proportional Hazards model, Kaplan-Meier analysis, and statistical tests including Student’s t-test were performed in R. Multiple hypothesis corrections were performed using the qvalue package [17]. All The Cancer Genome Atlas (TCGA) data were downloaded from the TCGA data portal using the published dataset. All TCGA data include primary ovarian tumors only. The Australian Oncology Group microarray data for ovarian tumors, GSE9891, was downloaded from GEO. Signals were normalized and determined by RMA [18] and only primary tumors were considered.

## Results

### Selection of cisplatin resistant OVCAR-8 ovarian cancer cells

OVCAR-8 cells were chosen for this study because they readily form spheroids [13], have features indicative of high-grade serous ovarian cancer [10], are mutant for p53 [19] and form xenografts with HGSOC histology [20]. Therefore, we hypothesized OVCAR-8 cells would provide a good cell line model to investigate cisplatin resistance mechanisms in spheroid conditions. When initially derived and tested OVCAR-8 cells did not significantly express metallothionein and were considered cisplatin sensitive relative to other patient derived cell lines [21]. Selection of drug resistant lines from increasing drug concentrations remains a powerful tool to gain insight into resistance mechanisms; however, little is understood with regard to resistance mechanisms in the context of spheroid culture systems, which appear to model tumors significantly better than monolayer culture [22].

**Fig 1:**
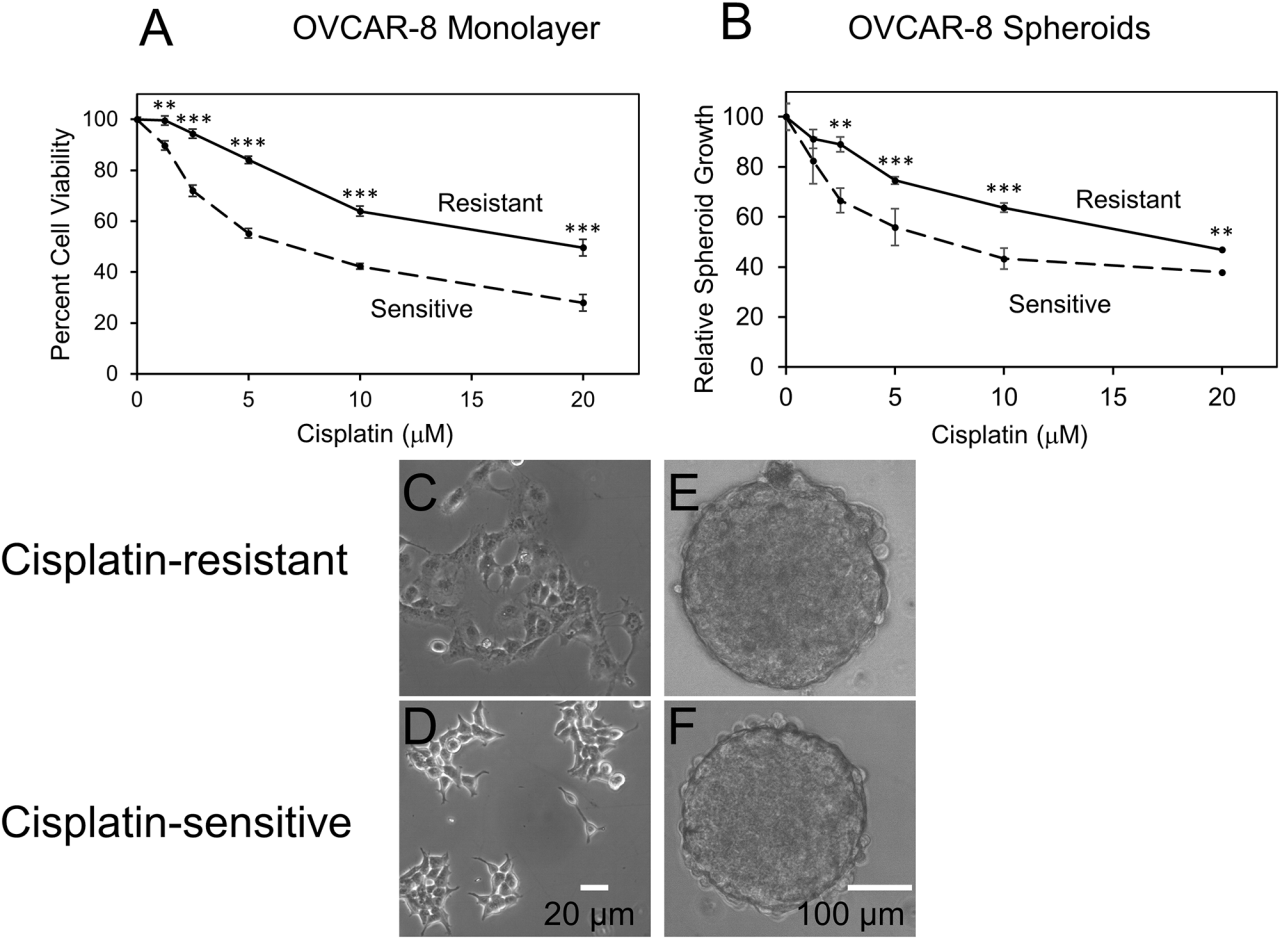
Selection of OVCAR-8 cells in increasing concentrations of cisplatin exposure leads to sustained resistance to cisplatin treatment. Cell viability of cisplatin resistant and sensitive cells grown as monolayers (A) or spheroids (B), expressed as a percentage of untreated viability, following exposure to varying concentrations of cisplatin for 96 h. Statistical comparisons were performed at each dose using a two-tailed Student’s t-test. Asterisks (**, p<0.01; ***, p<0.001) indicate significant difference in viability between cisplatin-sensitive (dashed line) and resistant cells (solid line) at the same cisplatin concentration (mean ± SE, n = 9). (C+D). Photomicrographs of cisplatin resistant (C) and sensitive (D) cells under phase contrast illumination. Resistant cells display stronger adhesion to the substrate while sensitive cells do not. Photomicrographs of spheroids from resistant (E) and sensitive (F) lines.

Serial exposure of OVCAR-8 cells to sub-lethal concentrations of cisplatin resulted in significant, lasting changes in cisplatin resistance (Fig 1). In both monolayer and spheroid conditions, cisplatin resistant cells, OVCAR-8R, showed a significant ~4-fold increase in the cisplatin IC50. In monolayer cultures, resistant cells showed greater adhesion to the culture vessel as indicated by the darker and flatter appearance under phase contrast (Fig 1C and 1D) although no morphological differences could be determined for the spheroids themselves (Fig 1E and 1F). The proliferation rate of resistant cells was also diminished compared to sensitive cells (data not shown). Cells remained resistant after growing in the absence of cisplatin for multiple generations. We hypothesized that the differences between the resistant and sensitive spheroids would better model mechanisms of resistance active in ovarian tumors compared to monolayer culture. To begin to test this hypothesis, we examined the mRNA expression differences between the parental OVCAR-8 and the resistant OVCAR-8R cells in spheroids using Affymetrix microarrays. OVCAR-8 and OVCAR-8R cells had strikingly different expression profiles with 3,139 transcripts significantly differentially expressed (Fc >2, q<0.01) (S2 Table).

**Fig 2:**
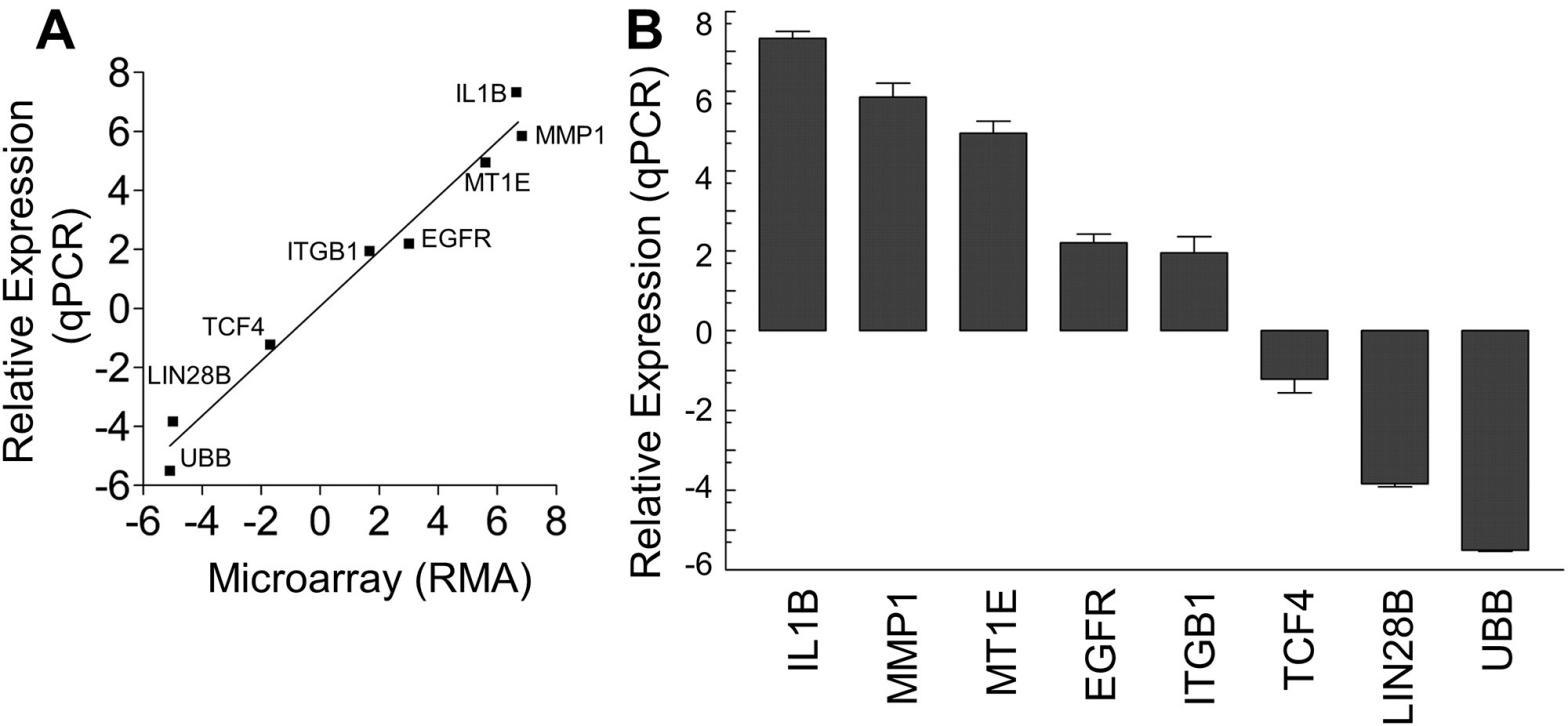
Differentially expressed transcripts between OVCAR-8R and OVCAR-8 spheroids are correlated between microarray and qPCR assays. Genes in the microarray dataset with differences in expression between sensitive and resistant ovarian cancer spheroids. (A) qPCR and microarray expression levels correlate. qPCR relative expression was calculated relative to GAPDH. Microarray scores are RMA. Trend line was determined by linear regression (r^2^ = 0.71, p = 0.009). (B) The fold changes for the selected transcripts.

Eight genes were selected with a wide range of expression differences between the resistant and sensitive cells to test in an orthogonal assay (Fig 2A and 2B). The differences in expression between OVCAR-8 and OVCAR-8R for these genes were significantly correlated between qPCR and microarray, supporting further analysis of the microarrays.

### OVCAR-8R cells sequester cisplatin

Drug transporters that mediate intracellular drug concentration have been investigated as drivers of chemoresistance [23]. Many candidate cisplatin exporters, associated with cisplatin sensitivity were significantly down-regulated, MDR1 (Fc > 3, q = 2e-5), hCTR1/SLC31A1 (Fc >2, q = 2.6e-4) [24], ATP7A (Fc>2, q = 2e-5) [25] while others were up-regulated, MRP1/ABCC1 (Fc = 3, q = 1.7e-5) [26] and ABCA3 (Fc>2, q = 2e-4) [27], in the resistant line (S2 Table). However, even though multiple pre-clinical models suggest the importance of MDR1 in cisplatin resistance, MDR1 expression has only rarely been correlated with responses and survival in ovarian cancer patients, even when considering multiple mechanisms of increasing MDR1 expression including promoter fusions [23].

The complicated mixture of differential expression of known cisplatin transporters did not immediately suggest that cisplatin transport was responsible for the observed resistance. To test whether drug transport and subsequent changes to intracellular concentration were driving resistance in OVCAR-8R spheroids, we measured intracellular platinum concentration in the spheroids using a cisplatin uptake assay by mass spectrometry. No difference in net uptake of cisplatin in cisplatin-sensitive (25.2 ± 8.2 μg cisplatin/g protein) spheroids, compared to cisplatin-resistant spheroids (26.5 ± 7.7 μg cisplatin/g protein) was observed. These observations indicate that even though expression of many drug transporters was down-regulated in resistant cells, the intracellular platinum concentration remained unaffected.

As OVCAR-8R spheroids did not demonstrate reduced intracellular platinum concentrations, other potential resistant pathways were evaluated. To handle intracellular platinum concentrations, sequestration can be mediated by platinum binding proteins including Glutathione-S Transferases and metallothioneins [5]. Large increases in expression of genes that bind cisplatin such as metallothioneins (MT2A and MT1E) were observed, indicating that the concept cisplatin may be sequestered in these cells. The metallothionein I and II isoforms have increased expression between 3-fold to 7-fold in OVCAR-8R cells, and MT1E was among the top genes up-regulated (Fc = 47, q = 3e-7) compared to OVCAR-8 cells. Higher expression of metallothioneins is a known mechanism of cisplatin resistance and increased expression in OVCAR-8R cells may drive platinum sequestration and drug resistance [4].

To identify pathways that may be mediating resistance, gene set enrichment analysis, (GSEA), was performed [28]. DNA repair pathways can be up-regulated in resistant cells and have been associated with patient survival [29]. The nucleotide excision repair pathway was not significantly differentially expressed (S3 Table). GSEA revealed that none of the major DNA repair pathways were significantly enriched in the OVCAR-8R spheroids (S3 Table). GSEA did identify numerous differentially expressed pathways (S3 Table), consistent with increased resistance including apoptosis regulation (Apoptosis Hallmark gene set NES = 1.9, FDR = 2e-4), including the apoptosis inhibitors, BIRC3 and BCL2L1 (S2 Table), and the inflammatory response Hallmark gene set (NES = 2.3, FDR < 1e-4), characterized by increased expression of IL6, IL18, IL8, IL1A, and TNF (S2 Table).

Reactome nucleotide metabolism gene set (NES = 1.4, FDR = 0.18) including NT5E, recently linked to cisplatin resistance [30] (S2 Table) was strongly up-regulated OVCAR-8R spheroids. Together, these observations indicate that multiple pathways are dysregulated contributing to the increased resistance of the OVCAR-8R spheroids.

### Resistant OVCAR-8R cells are more mesenchymal

**Fig 3:**
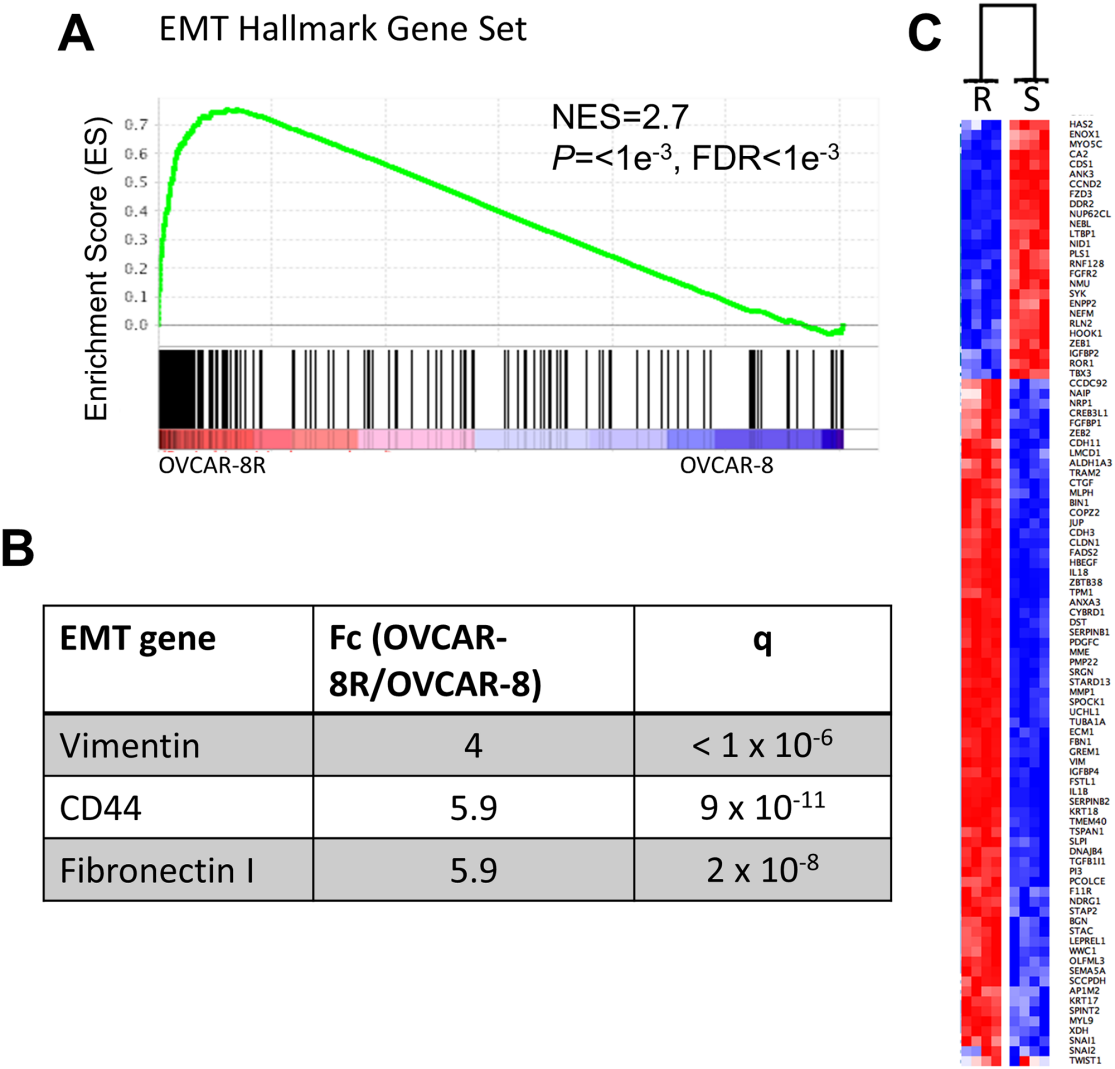
Cisplatin resistance genes significantly overlap with genes associated with the mesenchymal state. (A) GSEA enrichment shows that the EMT hallmark gene set is significantly biased towards OVCAR-8R spheroids compared to OVCAR-8. (B) Key mesenchymal markers are significantly up-regulated in OVCAR-8R compared to OVCAR-8 spheroids. (C) The Taube EMT signature separates the OVCAR-8R and OVCAR-8 spheroids by hierarchical clustering using Gene-E with Pearson correlation calculated distances. Each row represents a gene. R is the OVCAR-8R and S is the parental OVCAR-8 spheroids. Red indicates higher risk, blue indicates lower risk.

In order to test the hypothesis that the OVCAR-8R cells are more mesenchymal compared to OVCAR-8 cells, we evaluated whether the global gene expression program was indicative of a more mesenchymal phenotype. Mesenchymal cancer cells can be identified by examination of expression signatures indicative of mesenchymal states [31]. Epithelial-mesenchymal transition (EMT) status in ovarian tumors is typically associated with more aggressive tumors and shorter survival [32, 33]. The EMT hallmark gene set was the top-ranked cancer hallmark gene set when comparing OVCAR-8R and OVCAR-8 spheroids by GSEA (Fig 3A). Common mesenchymal markers including MMP1, CD44, TGFBI, FN1, and vimentin had significantly higher expression in OVCAR-8R cells (Fig 3B). Dr. Brugge and colleagues recently reported ovarian cancer cells that were more mesenchymal were more invasive and correlated with poor outcome [33]. We observed significant overlap between the 3,139 differentially expressed resistant genes and the EMT signature proposed by Taube et al. (2010) (Overlap = 91 genes, *P* = 0.0001). These 91 EMT genes were strongly differentially expressed between OVCAR-8 and OVCAR-8R spheroids (Fig 3C). However, none of the major EMT transcription factors (SNAI1, SNAI2, TWIST1, ZEB1, or ZEB2) were significantly up-regulated in OVCAR-8R cells. In fact, only ZEB1 was modestly differentially expressed and it was 2-fold down-regulated in the resistant cells. Of special note, the expression of these factors was already high in OVCAR-8 spheroids, likely from the adaptation to spheroid cultures, which has been reported to increase expression of mesenchymal markers [33].

Alternatively, EMT could be driven by YAP1, another transcriptional driver that increases cancer stemness and EMT in multiple systems [34]. YAP1 is expressed 3 fold higher in OVCAR-8R spheroids (Fc = 1.5, q = 1e-5) and a YAP1 gene expression program is up-regulated as well (YAP1_up gene set [35], NES = 1.75, FDR = 0.002). These observations indicate a potential mechanism of EMT control, independent of expression changes of more classical EMT transcription factors, as YAP1 has recently been reported to drive EMT in multiple systems including ovarian cancer [34, 36–38]. OVCAR-8 cells are KRAS mutant and activation of YAP1 may be working with KRAS activation to drive resistance [37], indicating that inhibition of both KRAS and YAP1 could overcome resistance in this model.

### Resistance gene expression signature is associated with survival

**Fig 4:**
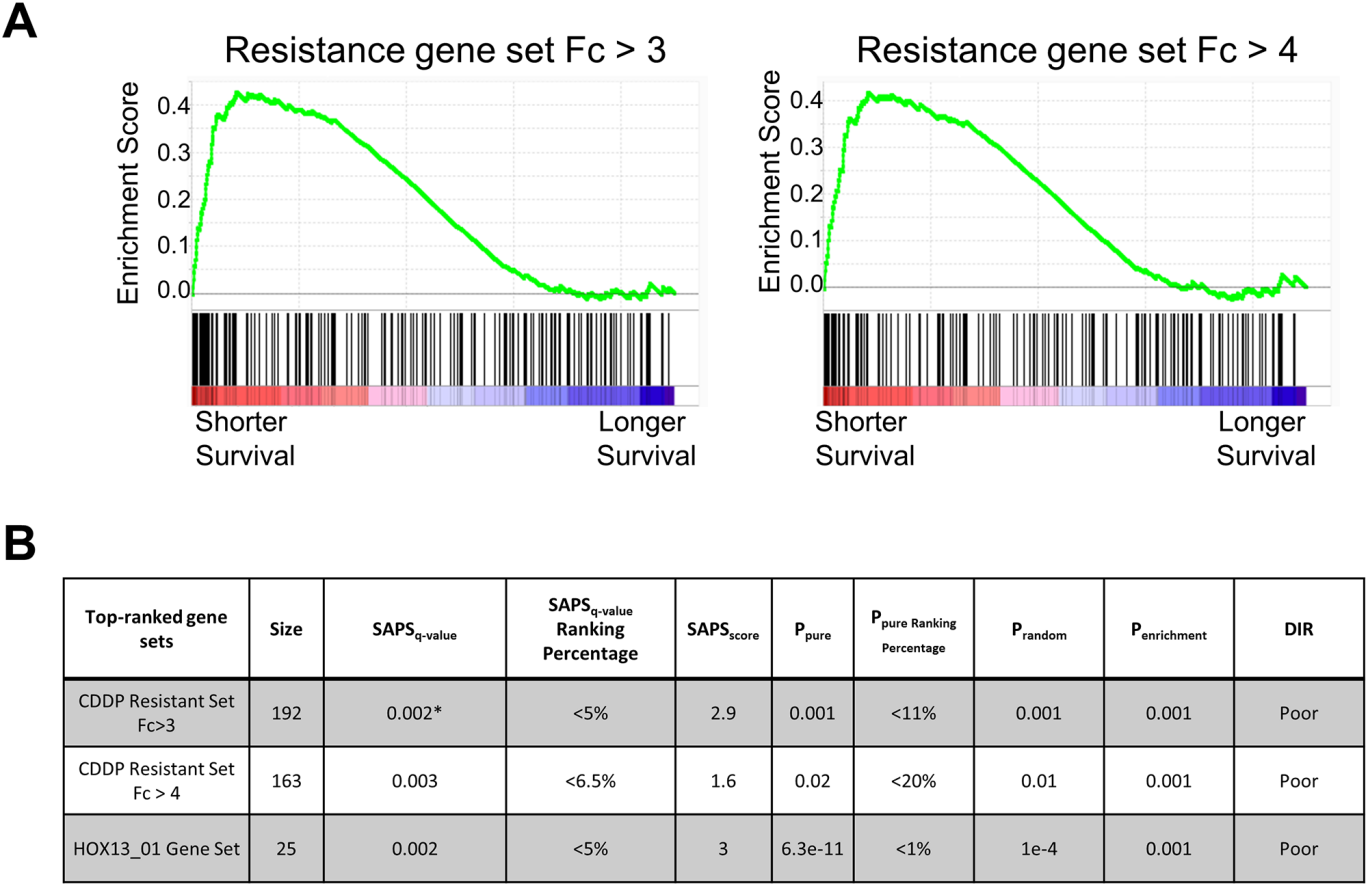
Differentially expressed transcripts are associated with survival across multiple ovarian cancer datasets. (A) SAPS analysis across 1735 ovarian tumor datasets suggests that both the Fc3 and Fc4 resistance transcript lists are significantly associated with shorter survival. (B) The Fc3 and Fc4 resistance gene sets were strongly enriched for shorter survival in the top 5% of all 5357 gene sets tested. Statistics for the resistance and representative strongly enriched gene sets are shown. The HOX13_01 gene set is shown as a positive control to compare the statistics.

An increased mesenchymal state has been associated with shorter survival in ovarian cancer [33]. In order to determine if mechanisms of resistance captured by gene expression in OVCAR-8R spheroids were relevant to ovarian tumors in patients, we pursued two approaches to examine gene expression in ovarian tumors. We tested if the cisplatin resistant gene sets were significantly associated with poor survival, relative to random gene sets and gene sets from MsigDB using the SAPS algorithm [39]. The SAPS approach considers a whole gene set, similar to GSEA, such that different combinations of transcripts may be biased in any one sample and the cumulative bias across all samples leads to the enrichment scores. The SAPS gene set approach was applied to 1735 ovarian cancer patients from twelve ovarian tumor datasets with available overall survival data as described [40]. SAPS compares each gene set to random gene sets of equivalent size (p_random, Fig 4). We examined two gene sets with the largest fold changes between the OVCAR-8R and OVCAR-8 with q<0.01 at Fc >3 and >4 and compared the resistance gene set to those from the Kegg, Reactome, BioCarta, and Gene Ontology databases. Fig 4 shows that these two gene sets rank among the highest of the 5373 gene sets tested, with q-values of 0.002 for Fc >3 and 0.003 for Fc >4, near the maximum obtainable by the algorithm. Fig 4A shows the strong enrichment of the resistance gene sets across all the ovarian tumors evaluated. As a control, we list the HOX13_01 gene set, which was reported to be one of the most significantly associated with survival in ovarian cancer [40]. These observations indicate that the most differentially expressed genes in the OVCAR-8R spheroids significantly identified patients with more aggressive and/or chemoresistant tumors leading to shorter overall survival.

**Fig 5:**
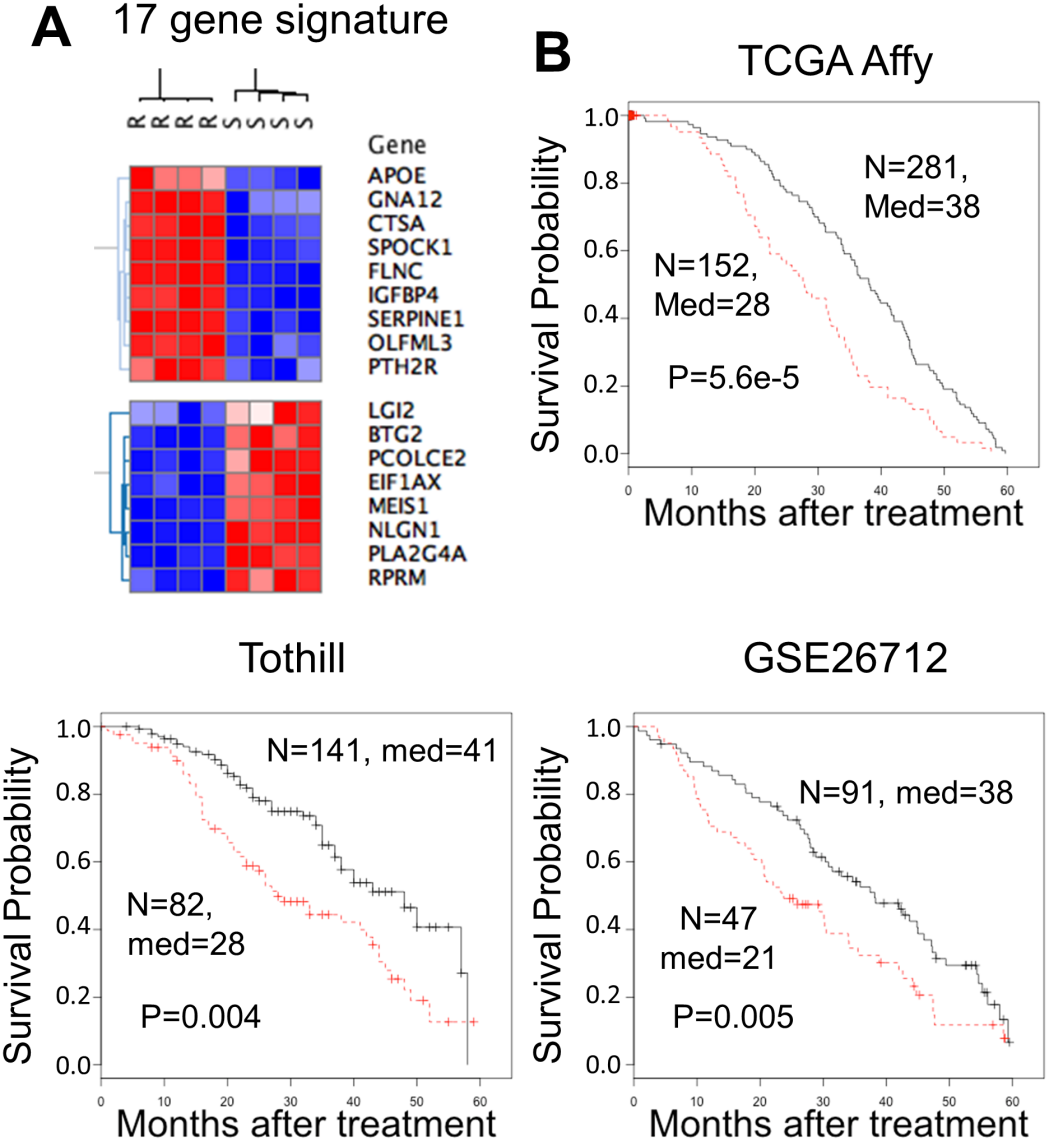
A 17 cisplatin resistance gene expression signature was associated with shorter survival across three independent datasets and distinguishes OVCAR-8R from OVCAR-8 cells. Genes up-regulated in OVCAR-8R spheroids are expressed higher in patients with shorter survival and vice-versa. (A) Hierarchical clustering with Pearson correlation distances separates high and low expression in OVCAR-8R and OVCAR-8 spheroids. Hierarchical clustering was performed using Gene-E software [16]. (B) Overall survival curves for the three indicated datasets. Only patients with survival data up to 5 years were considered. The number of patients (n), median survival (med), and the p-value from a log-rank test are indicated. The red line indicates high risk and the black line represents low risk patients.

To assess if a defined gene expression signature can be derived from the list of differentially expressed transcripts that is associated with patient survival, we identified a 17 gene expression signature that separated OVCAR-8 and OVCAR-8R cells (Fig 5A) and separated high and low risk patients in 3 independent datasets (Fig 5B). These 17 transcripts, selected from the 3,139 resistant gene expression list, were significantly associated with survival in all three independent datasets (Table 1). These observations suggest that features of the sensitive and resistant cells are indicative of tumor behavior in patients.

**Table 1:** The 17 transcripts constituting the resistance survival expression signature.

Gene Symbol	Gene Name	Expression Levels in High Risk Patients
APOE	apolipoprotein E	High
BTG2	BTG family, member 2	Low
CTSA	cathepsin A	High
EIF1AX	eukaryotic translation initiation factor 1A, X-linked	Low
FLNC	filamin C, gamma (actin binding protein 280)	High
GNA12	guanine nucleotide binding protein (G protein) alpha 12	High
IGFBP4	insulin-like growth factor binding protein 4	High
LGI2	leucine-rich repeat LGI family, member 2	High
MEIS1	Meis homeobox 1	Low
NLGN1	neuroligin 1	Low
OLFML3	olfactomedin-like 3	High
PCOLCE2	procollagen C-endopeptidase enhancer 2	High
PLA2G4A	phospholipase A2, group IVA (cytosolic, calcium-dependent)	Low
PTH2R	parathyroid hormone 2 receptor	High
RPRM	reprimo, TP53 dependent G2 arrest mediator candidate	Low
SERPINE1	serpin peptidase inhibitor, clade E (nexin, plasminogen activator inhibitor type 1), member 1	High
SPOCK1	sparc/osteonectin, cwcv and kazal-like domains proteoglycan (testican) 1	High

## Discussion

We developed a new spheroid model of drug resistance and provided evidence supporting its utility as a model for HGSOC tumors. We applied genome-wide expression profiling to gain insight into potential resistance mechanisms in the spheroids and examined which pathways may be relevant to patient tumors. In this model, resistance was not due to changes in drug transport or DNA repair, but rather to sequestration in combination with increased expression of anti-apoptosis pathways, cytokines, and an increased mesenchymal expression profile. Importantly, the changes adapted by the resistant cells in the expression profile identified patients with shorter survival and higher likelihood of relapse. We conclude that multiple mechanisms contribute to the cisplatin resistance of OVCAR-8R spheroids that are relevant to patients.

Previous *in vitro* models of selected cisplatin resistance in ovarian cancer cell lines revealed a plethora of resistance mechanisms [6], of which sequestration and platinum inactivation are examples [21, 41]. To determine how the OVCAR-8R spheroid resistance model compares to previously described cisplatin resistance models, we compared the gene expression changes to the A2780 resistance model in the GSE15709 [42] and GSE28648 [43] datasets. We found just 39 genes were differentially expressed across all 3 datasets (data not shown). We hypothesized that these 39 genes would be associated with survival and chemoresponse in patients. However, no significant association with survival was observed (data not shown). These findings indicate that the heterogeneity of the cell line models makes it challenging to connect gene expression signatures derived from such different conditions in this heterogeneous disease. This could be due to the strikingly different genetic backgrounds of these cell lines or because of the differences in monolayer vs. spheroid culture conditions. Phenotypic and genetic analysis of pre-clinical models, including established cell lines, has led to sometimes-conflicting interpretations of the validity of certain models [7]. The extreme genetic heterogeneity of ovarian tumors warrants continued development of pre-clinical models to capture the range of resistance mechanisms in various genetic backgrounds.

We specifically tested if resistance is associated with decreased platinum concentrations in the resistant cells and did not observe a significant difference in intracellular cisplatin concentrations. The gene expression data and platinum uptake assay were consistent with cisplatin resistance being caused by increased sequestration of platinum, through up-regulation of metallothionein and other sulfur rich proteins. Consistent with sequestration, metallothionein I and II isoforms were up-regulated between 3-fold to 7-fold in OVCAR-8R cells, and metallothionein-1E was among the top genes up-regulated (47-fold) compared to parental OVCAR-8 cells. Higher expression of metallothioneins is a known mechanism of cisplatin resistance and their increased expression in OVCAR-8R cells is likely contributing to the observed resistance [4]. Therefore, the role of transporters in this OVCAR-8R spheroid model are different than the OVCAR-8 derived cisplatin resistant NCI/ADR-RES line, characterized by high MDR1 expression [44].

The mesenchymal nature of ovarian cancer cells is most often associated with more drug resistant tumors and shorter survival [32, 33, 45, 46]. OVCAR-8 cells are an epithelial cell and 3D culture drives the mesenchymal state [47]. We observed increased expression in genes associated with epithelial—mesenchymal transition (EMT), such as YAP1, vimentin, fibronectin, collagen type 1 alpha 1, and P-cadherin expression [48]. OVCAR8 cells are reported to have relatively high YAP1 expression [49]. We observed changes in growth factor genes associated with EMT, such as transforming growth factor (TGF-β), epidermal growth factor (EGF), and fibroblast growth factor (FGF). The global gene expression pattern further supports the increased mesenchymal state of the OVCAR-8R spheroids. Together, these observations indicate that the OVCAR-8R spheroids model more mesenchymal, drug resistant tumors.

Our observations indicate that the OVCAR-8 spheroids represent a good model to examine cisplatin resistance *in vitro*. Similar to ovarian tumors, multiple mechanisms appear to contribute to resistance and the differentially expressed genes in the resistant cells correlate with poor outcomes in patients. These changes in gene expression were long-lasting responses as resistance was maintained after extensive culture in the absence of platinum. This *in vitro* model reflects similar cisplatin resistance mechanisms as those found in patients and will be useful for further physiological characterization of the resistance and investigation of methods for killing drug resistant cancer cells. We cannot conclude that all the observed changes are only observed in the spheroid form of the cells and not in the monolayer. Here, we focused on the spheroids and future efforts may evaluate differences in monolayer cultures.

A limitation of our study is that we only analyzed one cell line. Despite this limitation, this spheroid model is relevant to ovarian tumors as indicated by the common gene expression changes observed in the model and in ovarian tumors. The observation of the increased expression of many mesenchymal markers, a global gene expression profile associated with survival using a global analysis, SAPS, as well as the derivation of a specific 17 gene expression signature, all support the utility of this spheroid model to investigate mechanisms relevant to patients.

In summary, these observations indicate that the mechanisms of resistance in the OVCAR-8R cell line model are relevant to ovarian cancer patients, and support further investigation into the role of these genes in the development of resistance in ovarian cancer. This study of a spheroid model of ovarian cancer provides a foundation to gain new insights into cisplatin resistance in an *in vitro* model.

## Supporting Information

S1 Table List of primer sequences used for qPCR. (DOC)

S2 Table List of genes significantly differentially expressed between OVCAR-8R and OVCAR-8 spheroids. (XLSX)

S3 Table Gene Set Enrichment Analysis (GSEA) of the Cancer Hallmarks and Reactome gene sets. Four worksheets are provided listing the gene sets enriched in the resistant or parental cells.
(XLSX)
