# Supplementary material for: Cisplatin Resistant Spheroids Model Clinically Relevant Survival Mechanisms in Ovarian Tumors
Source: PLoS One. 2016 Mar 17;11(3):e0151089. doi: 10.1371/journal.pone.0151089 (PMC4795743; doi:10.1371/journal.pone.0151089)
Supplement: S1 Table — (DOC) [file pone.0151089.s001.doc]

| Gene | Forward primer (5’→3’) | Reverse primer (5’→3’) |
| --- | --- | --- |
| EGFR | CTGGAGAAAGGAGAACGCC | TCAACTCACGGAACTTTGGG |
| IL1B | ACAGTGGCAATGAGGATGAC | CGGAGATTCGTAGCTGGATG |
| ITGB1 | ATCCCTGAAAGTCCCAAGTG | GTCTACCAACACGCCCTTC |
| LIN28B | CAAAGGGAAGACACTACAGAAAAG | CTTCTTTGGCTGAGGAGGTAG |
| MMP1 | CTGCTTACGAATTTGCCGAC | GCCAAAGGAGCTGTAGATGTC |
| MT1E | CATCCCCTTTGCTCGAAATG | ACAGCAGCTCTTCTTGCAG |
| TCF4 | CTTCCTGTCCAGTCTGCG | ATCGGATTTGATCTCAGAGCTG |
| UBB | TTGGTGATTGGCAGGATCC | TTTCGATGGTGTCACTGGG |
| GAPDH | ACATCGCTCAGACACCATG | TGTAGTTGAGGTCAATGAAGGG |

**Table S1. List of primer sequences used for qPCR.**
